# Predicting 1-Year Renal Outcomes in Patients with Diabetic Kidney Disease in CKD Stages 3 to 4: A Multimodal Machine Learning Approach Fusing Clinical Composites and Pathology Images

**DOI:** 10.34133/research.1337

**Published:** 2026-06-30

**Authors:** Xiangmeng Li, Jinyu Liu, Erjina Huo, Peihua Zhang, Shimin Jiang, Cheng Zhou, Shunlai Shang, Wenge Li

**Affiliations:** ^1^ Department of Nephrology, China-Japan Friendship Hospital, Beijing, China.; ^2^ China-Japan Friendship Hospital, Chinese Academy of Medical Sciences and Peking Union Medical College, Beijing, China.; ^3^ Key Laboratory of Bone Metabolism and Physiology in Chronic Kidney Disease of Hebei Province, Affiliated Hospital of Hebei University, Baoding, Hebei, China.; ^4^ Capital Medical University, Beijing, China.; ^5^ University of Bristol, Bristol, UK.; ^6^ School of Big Data Science, Hebei Finance University, Baoding, Hebei, China.

## Abstract

Patients with diabetic kidney disease (DKD) at chronic kidney disease (CKD) stages 3 to 4 are at high risk for rapid renal function decline within 1 year. However, owing to the multifactorial complexity of the disease, effective prognostic tools that integrate multidimensional clinical and pathological information are currently lacking for this specific population. We conducted a retrospective cohort study involving 322 patients with biopsy-proven DKD (CKD stages 3 to 4) from the China-Japan Friendship Hospital and Hebei University Affiliated Hospital. Their clinical data and 2,576 renal biopsy pathology images were used to develop and validate a multimodal model. Machine learning was applied to integrate clinical composite indices and renal biopsy images to develop a prognostic prediction tool. Four key clinical predictors were identified: estimated glomerular filtration rate, 24-h urinary protein, systemic immune inflammation index, and estimated pulse wave velocity. Among the 6 machine learning algorithms used to develop the prediction models, the random forest algorithm achieved the best performance in the test set, with an area under the receiver operating characteristic (ROC) curve (AUC) of 0.889 and a precision–recall AUC (PR-AUC) of 0.921 for predicting the 1-year composite renal endpoint. The integration of pathological features led to a marked improvement in the performance of the model (ROC-AUC: 0.923 vs. 0.898). External validation demonstrated that incorporating pathological information into the model increased the ROC-AUC from 0.885—achieved when clinical composite indices alone were used as predictors—to 0.930. In this study, machine learning-based automated image analysis of glomerular crescent-shaped changes and renal interstitial fibrosis was integrated with established clinical composite indices to construct an accurate model for predicting short-term renal prognosis of DKD at CKD stages 3 to 4 and to provide a potential tool for improved risk stratification.

## Introduction

Diabetic kidney disease (DKD) has become a predominant cause of chronic kidney disease (CKD) and end-stage kidney disease [1]. Notably, upon progression to CKD stages 3 to 4, renal function deterioration accelerates substantially, with a substantial proportion of patients advancing to end-stage kidney disease (ESKD) requiring renal replacement therapy within 1 year. Early identification of high-risk individuals and timely intervention during this critical phase are essential. However, accurate short-term prognosis prediction in this population remains challenging due to dual limitations: existing models primarily rely on unidimensional clinical parameters, failing to capture the disease’s multifactorial complexity, while renal biopsy—often requiring laparoscopic-assisted specialized techniques—carries elevated risks and practical constraints, limiting both pathological data availability and clinical utility. These factors collectively hinder the development of effective prognostic tools, leaving a critical gap in risk stratification for this vulnerable cohort.

In recent years, machine learning has demonstrated important potential in disease prediction, capable of capturing complex nonlinear relationships among multidimensional clinical variables to provide superior risk stratification [2,3]. In particular, multimodal machine learning approaches enhance model robustness and predictive accuracy by integrating diverse data sources, such as clinical indicators, imaging, and pathological data. Deep learning techniques, notably convolutional neural networks (CNNs), have shown remarkable efficacy in processing high-dimensional data and have achieved substantial progress in automated analysis of renal biopsy pathology images [4], thereby offering a viable technical pathway for integrating clinical parameters with pathological information.

Despite advances in machine learning, current DKD prediction models exhibit limitations. Most rely predominantly on single biochemical parameters, focusing on diagnostic rather than prognostic prediction, thereby limiting their clinical translational value. Furthermore, although renal biopsy-derived pathological features—such as Kimmelstiel–Wilson (K-W) nodules, tubular atrophy, and interstitial fibrosis—hold important prognostic relevance, their systematic integration into predictive models remains inadequate due to procedural challenges. Notably, emerging composite indicators offer novel perspectives for risk assessment: the triglyceride–glucose index (TyG) reflecting insulin resistance, the atherogenic index of plasma (AIP) identifying lipid metabolism disorders, the systemic immune-inflammatory index representing inflammatory status, and estimated pulse wave velocity (ePWV) evaluating vascular stiffness [5–8]. These multidimensional parameters collectively delineate DKD progression trajectories through distinct pathophysiological mechanisms and have been independently associated with renal outcomes [9–11].

**Fig. 1.**
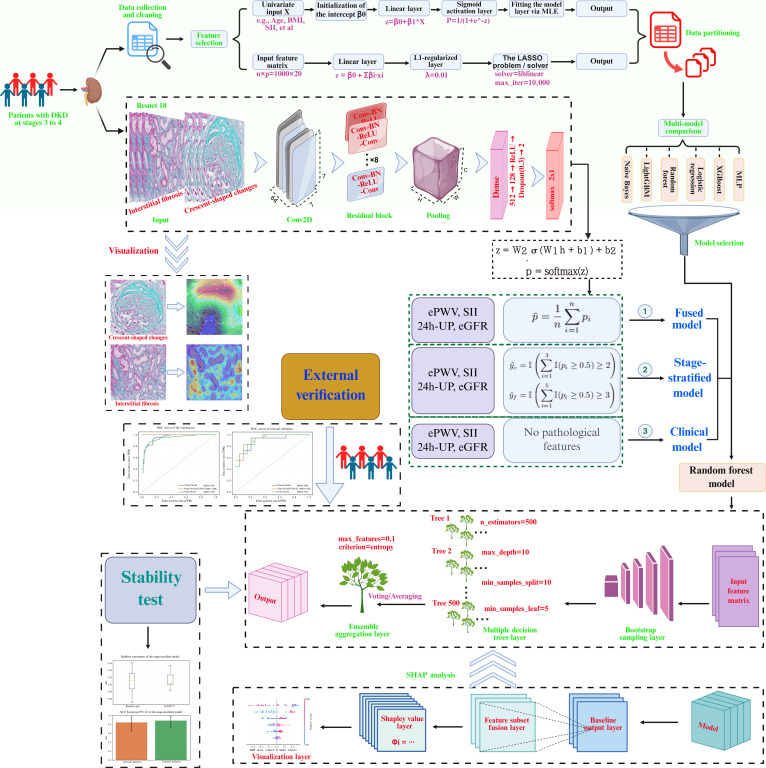
Framework overview. Development process of a multimodal predictive model for patients with DKD (CKD stages 3 to 4). Data collection and preprocessing: clinical data were cleaned, and key features were selected using methods such as univariate analysis and LASSO regression. Pathological images were processed using a ResNet18 encoder to extract features, generating probability scores for interstitial fibrosis and crescent formation. Model selection: multiple algorithms were compared, and the RF model was identified as the top-performing. Model construction: 3 distinct RF models were built, each with different input feature sets (X_clinical_ = [ePWV, SII, 24 h-UP, eGFR], X_stage-stratified_ = [$\hat{p}$c , $\hat{p}$f , ePWV, SII, 24 h-UP, eGFR], and X_fusion_ = [p-c , p-f , ePWV, SII, 24 h-UP, eGFR]). Evaluation and interpretation: the best-performing model was further subjected to external validation, SHAP analysis for interpretability, and stability testing. ePWV, estimated pulse wave velocity; SII, systemic immune inflammation index; 24 h-UP, 24-h urinary protein; eGFR, estimated glomerular filtration rate; RF, random forest.

Our study aims to develop a multimodal machine learning framework integrating CNN-based pathological feature extraction with multidimensional clinical indicators—including emerging composite biomarkers—to establish a prognostic prediction system for DKD patients at CKD stages 3 to 4 (Fig. 1). This pioneering approach systematically combines automated pathological image analysis with clinical composite indicators to overcome existing predictive limitations and enhance risk stratification for this high-risk population.

## Results

### Baseline characteristics

**Fig. 2.**
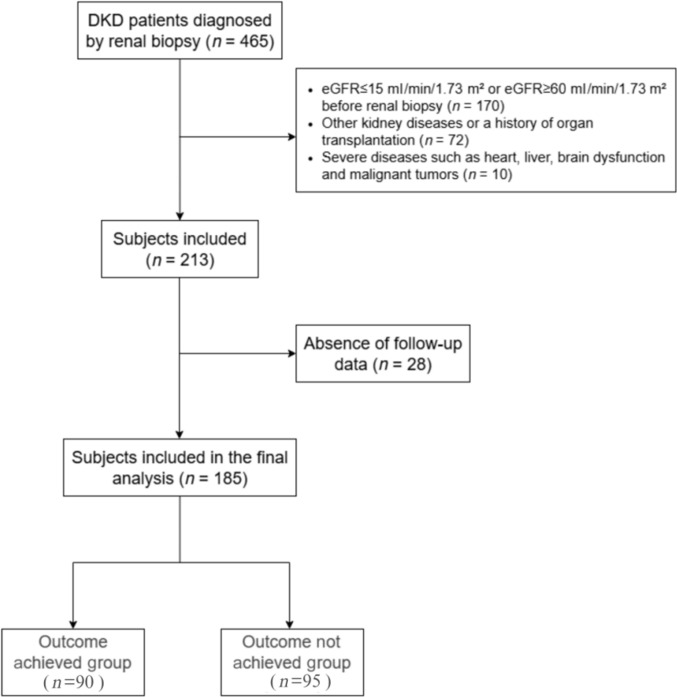
Patient enrollment flowchart.

A total of 185 patients were enrolled in this study (Fig. 2), with a mean age of 53.15 ± 9.59 years. Among them, 133 (71.9%) were male. The baseline demographic, clinical, and pathological characteristics of the patients at the time of renal biopsy are summarized in Table 1. Patients were divided into 2 groups based on whether they reached the composite kidney outcome after 1 year. Significant differences (*P* < 0.05) were observed between the 2 groups in terms of ePWV, systemic immune inflammation index (SII), estimated glomerular filtration rate (eGFR), 24-h urinary protein (24 h-UP), crescent-shaped changes, proportion of interstitial fibrosis, and degree of mesangial cell hyperplasia. However, no significant differences were found in age, sex, body mass index (BMI), AIP, TyG, or other pathological features (*P* > 0.05).

**Table 1.** Baseline characteristics of the enrolled patients

	Overall	Renal composite outcome		*P* value ^b^
	*n* = 185 ^a^	No, *n* = 95 ^a^	Yes, *n* = 90 ^a^	
Demographics				
Age	53.15 ± 9.59	53.24 ± 9.54	53.04 ± 9.69	0.89
Gender				0.69
Male	133 (71.9%)	70 (72.9%)	63 (70.0%)	
Female	52 (28.1%)	25 (26.3%)	27 (30.0%)	
BMI	26.01±3.56	26.28 ± 3.34	25.73 ± 3.77	0.29
Clinical history				
CAD				0.89
No	163 (88.11)	84 (88.42)	79 (87.78)	
Yes	22 (11.89)	11 (11.58)	11 (12.22)	
Stroke				0.56
No	161 (87.03)	84 (88.42)	77 (85.56)	
Yes	24 (12.97)	11 (11.58)	13 (14.44)	
DR				0.11
No	81 (43.78)	47 (49.47)	34 (37.78)	
Yes	104 (56.22)	48 (50.53)	56 (62.22)	
DFU				1.00
No	183 (98.92)	94 (98.95)	89 (98.89)	
Yes	2 (1.08)	1 (1.05)	1 (1.11)	
DPN				0.40
No	137 (74.05)	74 (77.89)	63 (70.00)	
Yes	45 (24.32)	19 (20.00)	26 (28.89)	
Clinical characteristic				
AIP	0.28 ± 0.29	0.30 ± 0.29	0.25 ± 0.28	0.25
TyG	9.33 ± 0.75	9.37 ± 0.80	9.29 ± 0.69	0.46
ePWV	9.38 ± 1.25	9.16 ± 1.19	9.62 ± 1.27	0.01
SII	649.73 (502.53–876.05)	604.19 (455.46–763.76)	721.04 (534.28–955.30)	<0.01
eGFR	44.63 (34.31–52.02)	50.08 (45.58–56.20)	36.62 (27.80–43.38)	<0.01
24 h-UP	5.15 (3.05–7.34)	3.27 (1.61–5.53)	6.51 (4.39–8.22)	<0.01
Pathological features				
K-W nodules present	88 (47.6%)	38 (40.0%)	50 (55.6%)	0.07
Crescent-shaped changes present	59 (31.9%)	11 (11.6%)	48 (53.3%)	<0.01
Capillary microaneurysms present	132 (71.4%)	67 (70.5%)	65 (72.2%)	0.93
Hyaline droplets present	120 (64.9%)	57 (60.0%)	63 (70.0%)	0.20
Cap lesion present	113 (61.1%)	52 (54.7%)	61 (67.8%)	0.10
Tubular atrophy^‡^				0.08
Absent	1 (0.5%)	1 (1.1%)	0 (0.0%)	
<25%	36 (19.5%)	24 (25.3%)	12 (13.3%)	
25%–50%	143 (77.3%)	69 (72.6%)	72 (82.2%)	
>50%	5 (2.7%)	1 (1.1%)	4 (4.4%)	
Interstitial fibrosis^‡^				<0.01
≤25%	87 (47.0%)	65 (68.4%)	22 (24.4%)	
>25%	98 (53.0%)	30 (31.6%)	68 (75.6%)	
Arteriolar hyalinosis^‡^				0.73
0	25 (13.5%)	14 (14.7%)	11 (12.2%)	
1	157 (84.9%)	80 (84.2%)	77 (85.6%)	
2	3 (1.6%)	1 (1.1%)	2 (2.2%)	
Mesangial expansion ^c^				<0.01
Mild	49 (26.5%)	35 (36.8%)	14 (15.6%)	
Severe	136 (73.5%)	60 (63.2%)	76 (84.4%)	

BMI, body mass index; CAD, coronary artery disease; DR, diabetic retinopathy; DFU, diabetic foot ulcer; DPN, diabetic peripheral neuropathy; AIP, atherogenic index of plasma; TyG, triglyceride–glucose index; ePWV, estimated pulse wave velocity; SII, systemic immune-inflammation index; eGFR, estimated glomerular filtration rate; 24 h-UP, 24-h urine protein; K-W nodules, Kimmelstiel–Wilson nodules

^a^ Mean ± SD or median [Q1 to Q3] for continuous variables and *n* (%) for categorical variables.

^b^ Between-group comparisons by *t* test, Mann–Whitney *U*, or *χ*^2^ as appropriate.

^c^ Pathological features were graded according to the scoring system of the Renal Pathology Society consensus classification [38].

### Feature selection

Univariate logistic regression analysis was performed, and the results are presented in Table 2. A total of 10 variables showed significant associations with the outcome (*P* ≤ 0.05), including K-W nodules (*P* = 0.03), crescent-shaped changes (*P* < 0.01), Cap lesion (*P* = 0.07), tubular atrophy (*P* = 0.01), interstitial fibrosis (*P* < 0.01), mesangial hypercellularity (*P* < 0.01), SII (*P* < 0.01), ePWV (*P* = 0.01), 24 h-UP (*P* < 0.01), and eGFR (*P* < 0.01). However, after adjustment for eGFR, Cap lesion (*P* = 0.46) and tubular atrophy (*P* = 0.87) were no longer significantly associated with the outcome and were therefore excluded. Finally, 8 variables—K-W nodules, crescent-shaped changes, degree of interstitial fibrosis, mesangial expansion, SII, ePWV, 24 h-UP, and eGFR—were selected for further screening.

**Table 2.** Results of the univariate logistic regression analysis

Variable	Unadjusted		Age adjusted		eGFR adjusted		Gender adjusted	
	OR (95% CI)	*P* value	OR (95% CI)	*P* value	OR (95% CI)	*P* value	OR (95% CI)	*P* value
Gender	0.83 (0.44–1.58)	0.58	0.83 (0.44–1.58)	0.57	0.76 (0.35–1.63)	0.48	Adjusted	Adjusted
K-W nodules	1.88 (1.05–3.36)	0.03	1.89 (1.05–3.40)	0.03	2.17 (1.06–4.42)	0.03	1.88 (1.05–3.36)	0.04
Crescent-shaped changes	8.73 (4.11–18.52)	<0.01	8.73 (4.11–18.52)	<0.01	9.22 (3.83–22.22)	<0.01	8.73 (4.11–18.52)	<0.01
Capillary microaneurysms	1.09 (0.57–2.06)	0.80	1.09 (0.57–2.06)	0.80	0.78 (0.36–1.69)	0.53	1.09 (0.57–2.06)	0.80
Hyaline droplets	1.56 (0.85–2.86)	0.16	1.56 (0.84–2.87)	0.16	1.17 (0.56–2.45)	0.67	1.56 (0.85–2.86)	0.16
Cap lesion	1.74 (0.96–3.17)	0.07	1.74 (0.96–3.17)	0.07	1.31 (0.64–2.71)	0.46	1.74 (0.96–3.17)	0.07
Tubular atrophy	2.40 (1.21–4.77)	0.01	2.42 (1.21–4.83)	0.01	0.93 (0.41–2.10)	0.87	2.40 (1.21–4.77)	0.01
Interstitial fibrosis	6.70 (3.51–12.79)	<0.01	6.71 (3.51–12.82)	<0.01	5.93 (2.76–12.77)	<0.01	6.70 (3.51–12.79)	<0.01
Arteriolar hyalinosis	1.31 (0.60–2.87)	0.50	1.32 (0.60–2.91)	0.49	1.39 (0.53–3.63)	0.50	1.31 (0.60–2.87)	0.50
Mesangial expansion	3.17 (1.56–6.42)	<0.01	3.19 (1.57–6.48)	<0.01	2.48 (1.08–5.72)	0.03	3.17 (1.56–6.42)	<0.01
Age	1.00 (0.97–1.03)	0.89	Adjusted	Adjusted	1.00 (0.97–1.04)	0.94	1.00 (0.97–1.03)	0.89
BMI	0.96 (0.88–1.04)	0.29	0.95 (0.88–1.04)	0.27	0.96 (0.87–1.06)	0.39	0.96 (0.88–1.04)	0.29
AIP	0.55 (0.20–1.53)	0.25	0.55 (0.20–1.53)	0.25	0.39 (0.11–1.38)	0.14	0.55 (0.20–1.53)	0.25
TyG	0.86 (0.59–1.27)	0.46	0.86 (0.59–1.27)	0.46	0.83 (0.52–1.32)	0.43	0.86 (0.59–1.27)	0.46
SII	1.00 (1.00–1.00)	<0.01	1.00 (1.00–1.00)	<0.01	1.00 (1.00–1.00)	0.01	1.00 (1.00–1.00)	<0.01
ePWV	1.37 (1.07–1.74)	0.01	3.07 (1.81–5.22)	<0.01	1.36 (1.01–1.83)	0.05	1.37 (1.07–1.74)	0.01
24 h-UP	1.51 (1.32–1.72)	<0.01	1.52 (1.32–1.74)	<0.01	1.44 (1.23–1.67)	<0.01	1.51 (1.32–1.72)	<0.01
eGFR	0.88 (0.85–0.91)	<0.01	0.88 (0.85–0.91)	<0.01	Adjusted	Adjusted	0.88 (0.85–0.91)	<0.01

K-W nodules, Kimmelstiel–Wilson nodules; BMI, body mass index; AIP, atherogenic index of plasma; TyG, triglyceride-glucose index; SII, systemic immune-inflammation index; ePWV, estimated pulse wave velocity; 24 h-UP, 24-h urine protein; eGFR, estimated glomerular filtration rate

**Fig. 3.**
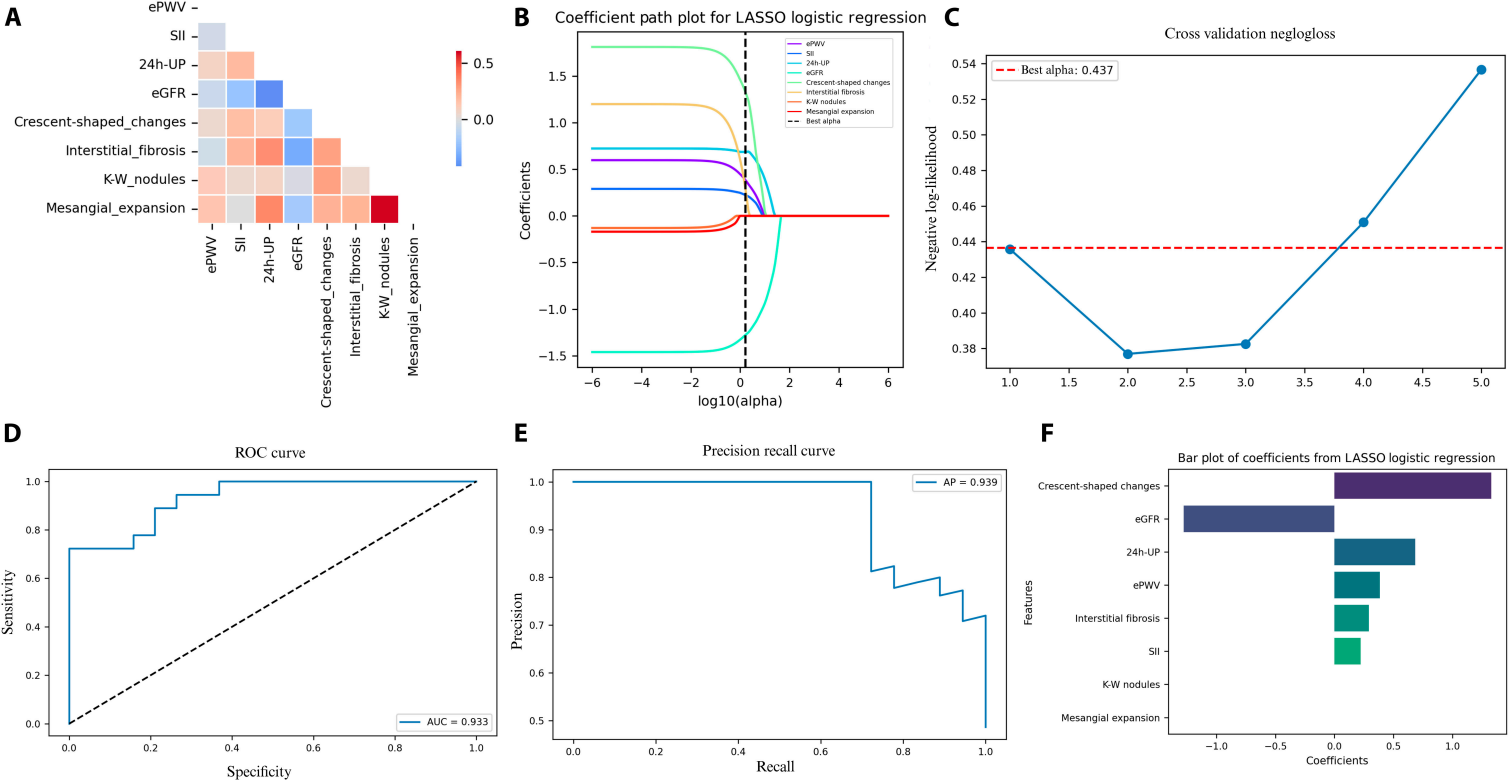
Variable selection in the LASSO logistic regression model. (A) Feature correlation heatmap. Red indicates a positive correlation, and blue indicates a negative correlation. (B) Coefficient path plot for the LASSO logistic regression. The vertical black dashed line marks the optimal regularization parameter (*α*). (C) Cross-validation performance curve for LASSO regression. The curve exhibits a characteristic “U-shape”. At *α* = 0.437, the model achieves its best balance between fitting ability and generalization performance. (D and E) ROC curve and PR curve. The AUC values were 0.933 and 0.939, respectively, demonstrating stable and reliable model performance. (F) Coefficient bar plot of the LASSO logistic regression. The coefficients for K-W nodules and mesangial expansion are zero, indicating that these features do not contribute predictive value. 24 h-UP, 24-h urine protein; ePWV, estimated pulse wave velocity; SII, systemic immune-inflammation index; eGFR, estimated glomerular filtration rate; K-W nodules, Kimmelstiel–Wilson nodules; ROC, receiver operating characteristic; PR, precision–recall; AUC, area under the curve.

A Least Absolute Shrinkage and Selection Operator (LASSO) logistic regression model was constructed. The optimal alpha (*α*) value of 0.437 was determined through 5-fold cross-validation, as shown in Fig. 3. Six variables were ultimately retained in the model: crescent-shaped changes (*β* = 1.33), 24 h-UP (*β* = 0.69), interstitial fibrosis (*β* = 0.29), eGFR (*β* = −1.28), SII (*β* = 0.22), and ePWV (*β* = 0.38).

### Comparison of multiple models

**Fig. 4.**
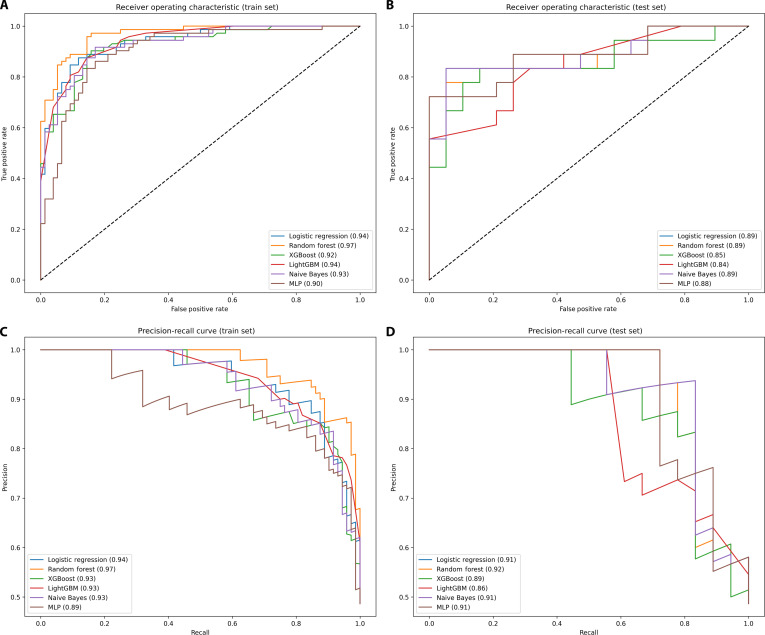
Comparison of multiple machine learning models. (A) ROC curves of different models (training set). (B) ROC curves of different models (test set). (C) PR curves of different models (training set). (D) PR curves of different models (test set). ROC, receiver operating characteristic; PR, precision–recall.

Utilizing the selected features, prediction models were constructed using 6 different machine learning algorithms. Among all models evaluated, the random forest (RF) model demonstrated the best discriminative ability in the test set, with an area under the receiver operating characteristic (ROC) curve (AUC) of 0.967 and a precision–recall AUC (PR-AUC) of 0.967. Its performance remained comparable to that observed in the training set (ROC-AUC = 0.889, PR-AUC = 0.921), indicating strong and stable predictive performance (Fig. 4). Consequently, the RF algorithm was selected to establish the final prediction model (Table 3).

**Table 3.** Performance comparison of the 6 machine learning models

Model	Train accuracy	Train precision	Train recall	Train F1	Train ROC-AUC	Train PR-AUC
Logistic regression	0.851	0.917	0.764	0.833	0.937	0.939
Random forest	0.878	0.935	0.806	0.866	0.967	0.967
XGBoost	0.838	0.875	0.778	0.824	0.925	0.928
LightGBM	0.851	0.868	0.819	0.843	0.941	0.928
Naive Bayes	0.831	0.885	0.750	0.812	0.930	0.934
MLP	0.736	0.884	0.528	0.661	0.902	0.889
Model	Test accuracy	Test precision	Test recall	Test F1	Test ROC-AUC	Test PR-AUC
Logistic regression	0.892	0.938	0.833	0.882	0.886	0.914
Random forest	0.865	0.933	0.778	0.848	0.889	0.921
XGBoost	0.838	0.833	0.833	0.833	0.854	0.886
LightGBM	0.757	0.714	0.833	0.769	0.844	0.857
Naive Bayes	0.892	0.938	0.833	0.882	0.886	0.914
MLP	0.865	1.000	0.722	0.839	0.883	0.913

XGBoost, eXtreme Gradient Boosting; MLP, multilayer perceptron; AUC, area under the curve; PR-AUC, precision–recall AUC

### Model development and validation

**Fig. 5.**
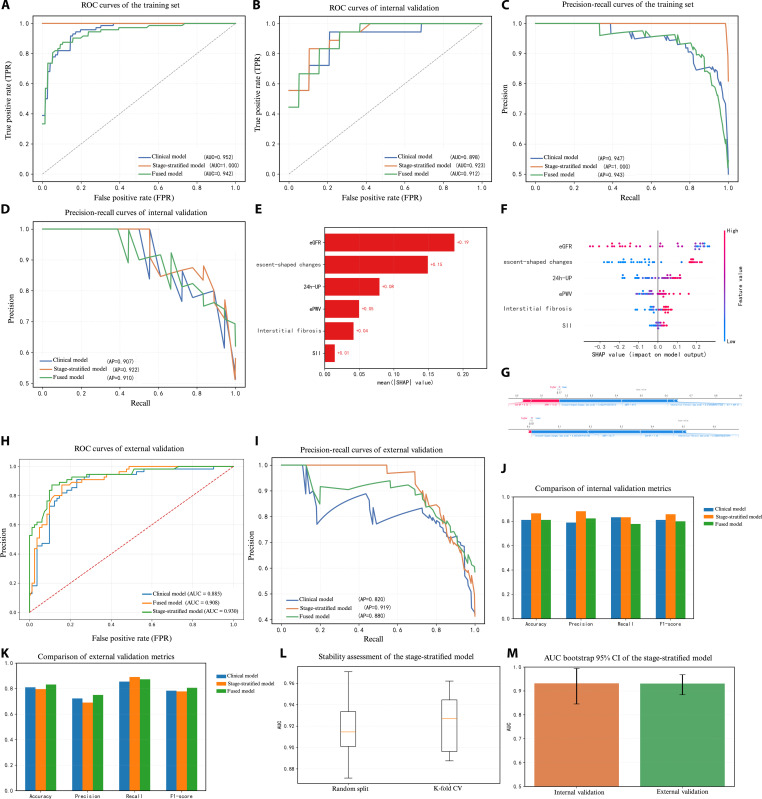
Development and validation of multimodal models for predicting 1-year renal outcomes in patients with stage 3 to 4 CKD. This figure comprehensively illustrates the performance comparison and interpretability analysis of 3 models—the clinical model, the stage-stratified model, and the fused model—across the training, internal validation, and external validation phases. The stage-stratified model demonstrates optimal performance across predictive accuracy, generalization, interpretability, and stability, and is therefore recommended as the preferred multimodal model for predicting 1-year renal outcomes in this patient population. The individual panels are detailed as follows: (A) ROC curves on the training set. (B) ROC curves on the internal validation set. (C) Precision–recall curves on the training set. (D) Precision–recall curves on the internal validation set. (E) SHAP summary plot for the stage-stratified model, ranking feature importance. (F) SHAP beeswarm plot for the stage-stratified model, illustrating the directional relationship between feature values and predicted outcomes. (G) Individual SHAP explanation plots showing the direction and magnitude of each feature’s contribution to the prediction for specific samples (features are sorted in descending order of SHAP value; positive values push the prediction toward higher risk, negative values toward lower risk). (H) ROC curves on the external validation set. (I) Precision–recall curves on the external validation set. (J) Comparison of internal validation metrics. (K) Comparison of external validation metrics. (L) Box plot of model stability assessed by K-fold cross-validation. (M) Bootstrap-derived 95% confidence interval plot. ROC, receiver operating characteristic; SHAP, Shapley additive explanation.

All 3 models demonstrated strong discriminative performance in both the training and internal validation sets. The stage-stratified model achieved the highest AUC values (training set AUC = 1.000; validation set AUC = 0.923), representing the best overall performance. This was followed by the fusion model (training set AUC = 0.942; validation set AUC = 0.912), whereas the clinical model exhibited lower predictive ability (training set AUC = 0.952; validation set AUC = 0.898). These results indicate that pathological image information provides marked complementary value in predicting 1-year renal outcomes for DKD among patients with stage 3 to 4 CKD, as shown in Fig. 5A and B. The superior performance of the stage-stratified model over the fusion model suggests that the multivoting strategy for determining pathological lesion grades is more effective than averaging computed probabilities from image features. This discrepancy may stem from variations in tissue staining intensity in practice, which can arise from repeated use of staining reagents. Based on the shape of the internal validation curves, the stage-stratified model maintained a high true-positive rate even at low false-positive rates, indicating strong sensitivity under stringent threshold settings. Meanwhile, the fusion model converged with the stage-stratified model at moderate-to-high false-positive rates, suggesting that pathological probability continues to provide marginal gains in discrimination. PR curves and the PR-AUC metric focus more on the model’s ability to detect positive samples. The stage-stratified model consistently outperformed both the fusion model and the clinical model across the training and internal validation sets (PR-AUC: 0.922 vs. 0.910 vs. 0.907), suggesting that pathological image information provides greater added value for identifying positive cases under imbalanced data conditions, as shown in Fig. 5C and D.

**Fig. 6.**
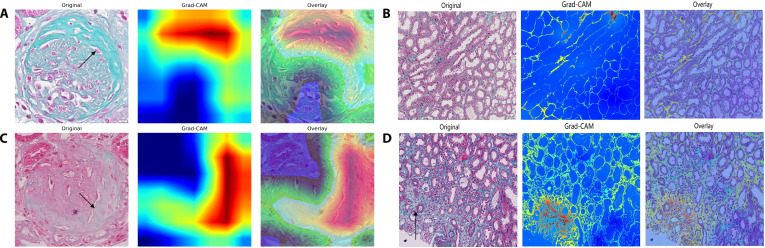
Heatmap for feature visualization of image recognition in multimodal models. (A and C) Crescent-shaped changes within the glomeruli are indicated by black arrows. (B and D) Black arrows denote interstitial inflammatory cell infiltration, tubular atrophy, and progressive fibrotic replacement in the tubulointerstitium.

This multimodal model integrates image and laboratory indicators. Its image component, trained on 2,576 renal biopsy images, extracts pathological features. The model’s attention was visualized using heatmapping techniques, which revealed that its focus areas consistently aligned with regions of known pathological lesions, thereby validating both its interpretability and clinical relevance. These results are illustrated in Fig. 6.

### Shapley additive explanations analysis

Shapley additive explanations (SHAP) analysis revealed that both pathological predictions and clinical composite indicators contributed to the predictions in the stage-stratified model. Features were ranked by their mean absolute SHAP values in descending order: eGFR (mean |SHAP| = 0.19) was the most important predictor, followed by crescent-shaped changes (mean |SHAP| = 0.15), 24 h-UP (mean |SHAP| = 0.08), ePWV (mean |SHAP| = 0.05), interstitial fibrosis (mean |SHAP| = 0.04), and SII (mean |SHAP| = 0.01) (shown in Fig. 5E). The beeswarm plot further illustrates the directional relationship between feature values and predicted outcomes: lower eGFR was associated with a higher risk of the endpoint event, whereas higher values of crescent-shaped changes, 24 h-UP, ePWV, interstitial fibrosis, and SII were linked to an increased event risk (shown in Fig. 5F). From the overall importance ranking, both pathological probability features and core clinical indicators collectively formed the primary basis for discrimination, suggesting that the model does not rely solely on a single source of information but instead integrates multidimensional clues to form risk assessments. Subsequently, the model randomly generated individualized explanation plots for 2 samples. These plots illustrate the direction and magnitude of each feature’s contribution to the prediction for individual cases, highlighting that the model’s decision stems from the cumulative effect of multiple features and aiding in understanding case-specific risk sources. The individual explanation plots further show that the dominant factors differ across patients, indicating that the model dynamically weights pathological information and clinical status based on the individual case, reflecting the inherent heterogeneity observed in clinical practice (shown in Fig. 5G).

### External validation

In the external validation, the stage-stratified model was markedly superior to the fusion model (AUC = 0.930 vs. 0.908), and both outperformed the clinical model (AUC = 0.885), suggesting that pathological image features contribute certain discriminative value. In both internal and external validation, AUC values of the stage-stratified model were 0.923 and 0.930, showing no marked decline and even a slight improvement in external validation. This indicates that the model possesses excellent generalization capability and robustness, without overfitting. From the PR curve perspective, a consistent performance hierarchy was observed: the stage-stratified model demonstrated superior predictive utility compared to both the fusion model and the clinical model, with respective PR-AUC values of 0.919, 0.880, and 0.820. Notably, the stage-stratified model maintained excellent discriminative performance in external validation compared to internal validation (PR-AUC: 0.919 vs. 0.922). The PR curve displayed a smooth morphology without erratic fluctuations, and no degradation in performance was observed between validation cohorts, collectively indicating robust generalization capability and an absence of overfitting (shown in Fig. 5H and I). A comparative analysis of the 3 models across internal and external validation sets—evaluated on accuracy, precision, recall, and F1-score—reveals that the stage-stratified model exhibited balanced performance in internal validation. In external validation, it demonstrated notably strong performance in recall, indicating its clinical utility in effectively minimizing missed diagnoses and underscoring its potential for real-world application (shown in Fig. 5J and K and Table 4).

**Table 4.** Performance of the 3 models in internal and external validation

Model	Item	Accuracy	Precision	Recall	F1	ROC-AUC	PR-AUC
Clinical model	Internal validation	0.811	0.789	0.833	0.811	0.898	0.907
	External validation	0.810	0.723	0.855	0.783	0.885	0.820
Stage-stratified model	Internal validation	0.865	0.882	0.833	0.857	0.923	0.922
	External validation	0.796	0.690	0.891	0.778	0.930	0.919
Fused model	Internal validation	0.811	0.824	0.778	0.800	0.912	0.910
	External validation	0.832	0.75	0.873	0.807	0.908	0.880

AUC, area under the receiver operating characteristic curve; PR-AUC, precision–recall AUC

### Stability assessment of the stage-stratified model

The random split method yielded an AUC of 0.917 ± 0.026, while cross-validation produced an AUC of 0.923 ± 0.032. Bootstrap analysis showed that the stage-stratified model achieved an AUC of 0.932 (95% CI [0.845 to 0.994]) in internal validation and 0.931 (95% CI [0.884 to 0.968]) in external validation. The boxplot derived from K-fold cross-validation exhibits a narrower interquartile range compared to that from random split validation, indicating that performance estimates obtained through repeated data partitioning are more robust and less susceptible to the variability of a single split. The median AUC values under both methods remained closely aligned, with no abrupt decline observed, which provides strong evidence that the model’s performance is reliable and not attributable to overfitting on a particular data partition. Consequently, both the stability assessment boxplot and the bootstrap 95% confidence interval (CI) plot confirm that the stage-stratified model achieves a consistently high AUC with stable and reproducible results (shown in Fig. 5L and M).

## Discussion

Currently, the internationally recognized health outcome prediction models for diabetes complications with strong validation primarily include RECODe (risk equations for complications of type 2 diabetes) [12] and UKPDS-OM2 (United Kingdom prospective diabetes study outcomes model version 2) [13], both of which generally cover major endpoints such as cardiovascular, cerebrovascular, and renal outcomes. On this basis, Quan et al. [14] developed the CHIME (Chinese Hong Kong integrated modeling and evaluation) model targeting the Asian population. However, these models commonly suffer from overly broad coverage and a lack of specificity for patients with advanced DKD. Most existing models focus on screening early-stage DKD risk [15–17] or cover the full spectrum of CKD, lacking refined stratification tailored specifically to patients with CKD stages 3 to 4. Consequently, effective prognostic prediction tools for DKD patients who have progressed to CKD stages 3 to 4 and are at risk of rapid renal function decline are still lacking. Zou et al. [18] developed a risk prediction model for progression to ESKD in type 2 diabetes-related DKD, incorporating 5 key predictors (cystatin C, serum albumin, Hb, 24h-UTP, and eGFR), with the RF model achieving an AUC of 0.90. Our study similarly found that the RF algorithm excels at predicting renal function progression: using only clinical indicators, the AUC was 0.898; after incorporating renal biopsy pathological features, the AUC markedly improved to 0.923 and, in external validation, reached 0.930. This study is the first to adopt a multimodal prediction model strategy integrating pathological features. Notably, this study does not advocate routine kidney biopsy for all patients with CKD stage 3 to 4 DKD. Instead, it focuses on a specific population whose renal pathological data have already been obtained for clinical indications. With the increasing maturity and minimal invasiveness of percutaneous renal biopsy techniques, patients with CKD stage 3 to 4 (eGFR 15 to 60 ml/min) can now safely undergo biopsy via routine minimally invasive approaches in most clinical settings, thereby enhancing the feasibility of implementing our model in real-world practice. Currently, pathological findings in these patients are primarily used for etiological diagnosis, while their prognostic potential remains largely untapped. We selected the 1-year prediction endpoint based on the following considerations: First, we specifically focused on patients with CKD stages 3 to 4 who experience composite renal endpoints within 1 year, aiming to identify individuals at high risk of rapid progression. In real-world clinical practice, these patients experience high rates of hospital readmission and frequently present with severe heart failure, pronounced edema, and an increased risk of serious acid–base disturbances, electrolyte imbalances, and even sudden cardiac death. Early identification of this high-risk population enables timely implementation of more intensive supportive care, which may reduce hospitalization risk and delay dialysis initiation. Second, pathological imaging features—such as crescent formation and interstitial fibrosis—are more strongly associated with short-term renal function decline, making them more suitable as short-term prognostic biomarkers.

From the perspective of clinical indicators, the variables incorporated in the model reveal potential driving mechanisms behind the rapid progression of DKD. Both eGFR and 24 h-UP quantification are key indicators recommended by the Kidney Disease Improving Global Outcomes guidelines for assessing DKD progression. In this study, eGFR demonstrated higher predictive value than urinary protein, which aligns with the pathophysiological evolution across disease stages: proteinuria serves primarily as a key predictor in early-stage CKD [19], whereas in CKD stages 3 to 4, dynamic changes in eGFR more comprehensively reflect renal function status and complication risks [20], thereby offering greater guidance for risk stratification and treatment decisions in mid-to-late stage patients. The SII, which integrates platelet, neutrophil, and lymphocyte counts, provides a comprehensive reflection of the body’s inflammatory and immune status. In the context of DKD, an elevated SII not only holds diagnostic value [11] but also is closely associated with disease incidence, severity, and risks of all-cause and cardiovascular mortality [21,22]. On the other hand, hyperglycemia-induced endothelial injury and microvascular remodeling promote arterial stiffness, further impairing renal autoregulation and accelerating renal function decline [23]. The ePWV, a widely recognized indicator of arterial stiffness calculated based on age and blood pressure, is closely linked to metabolic diseases and cardiovascular events [24–26]. Studies have shown its independent association with the prevalence of DKD [27], and it is higher in CKD populations compared to healthy controls [28], underscoring its important potential as a predictor of renal outcomes.

Furthermore, renal pathological features hold irreplaceable value in prognostic assessment. Renal interstitial fibrosis and glomerular crescent-shaped changes have been established as key histological predictors of DKD progression. Stefan et al. [29] reported that each 1-grade increase in the interstitial fibrosis and tubular atrophy score markedly elevated the risk of ESKD or renal replacement therapy (HR 1.58, 95% CI 1.00 to 2.54), an association that persisted even after adjustment for clinical variables including eGFR and proteinuria. Hu et al. [30] further demonstrated that incorporating interstitial fibrosis and tubular atrophy into prediction models improved the predictive performance for ESKD, with 2-year/5-year AUCs reaching 0.809 and 0.751, respectively. Since Elfenbein and Reyes [31] first described crescentic lesions in DKD, Mottl et al. [32] subsequently identified them as an independent predictor of ESKD in DKD patients with crescents. Subsequent studies, including those by Sun et al. and a recent Korean multicenter investigation by Bae et al. [33,34], have consistently validated crescentic lesions as an independent risk factor for the progression of DKD.

This study has limitations. First, while its retrospective design and the potential for bias due to missing data and loss to follow-up are inherent concerns, we partially addressed these by extending the inclusion period and increasing the sample size, which improved data completeness and cohort representativeness to meet basic model development requirements. Future studies should focus on external validation using multicenter or large-scale prospective cohorts to assess generalizability and robustness. Second, to ensure that the SII reliably reflects the microinflammatory state of DKD, we excluded patients with active infection (procalcitonin above the normal range), autoimmune diseases, tumors, or hematologic disorders. Consequently, our model is not applicable to these excluded populations, which represents another limitation of this study. In addition, incorporating novel predictors such as multi-omics biomarkers along with more advanced modeling techniques may further enhance model performance and advance personalized prognostic assessment.

## Conclusion

In this study, machine learning-based automated image analysis of glomerular crescent-shaped changes and renal interstitial fibrosis was integrated with established clinical composite indices to construct an accurate model for predicting short-term renal prognosis of DKD at CKD stages 3 to 4 and to provide a potential tool for improved risk stratification.

## Materials and Methods

### Study population

Patients who were diagnosed with DKD by renal biopsy in the Department of Nephrology at the China-Japan Friendship Hospital between 2004 July 1 and 2024 June 30 were enrolled in this study. Inclusion criteria were as follows: (a) age between 18 and 75 years; (b) diagnosis of type 2 diabetes mellitus according to the American Diabetes Association 2024 Standards of Care in Diabetes; (c) pathological confirmation of DKD by renal biopsy; and (d) an eGFR ranging from 15 to 60 ml/min/1.73 m^2^ at the time of renal biopsy. Exclusion criteria were as follows: (a) the presence of other renal diseases or a history of organ transplantation; (b) the presence of active infection (with procalcitonin levels exceeding the normal range); (c) the presence of severe cardiac, hepatic, or cerebral dysfunction; (d) the presence of malignancy, autoimmune diseases, tumors, or hematologic disorders; and (e) a follow-up duration of less than 1 year or incomplete baseline/follow-up data. This study was approved by the Ethics Committee of the China-Japan Friendship Hospital (Approval No. 2024-KY-129).

### Data collection

The data from a total of 185 patients were used for model training and internal validation. The following data were collected at the time of renal biopsy:

• Demographic characteristics: sex, age, and BMI.
• Clinical history: history of coronary artery disease, history of stroke, history of diabetic retinopathy, history of diabetic foot ulcer, and history of diabetic peripheral neuropathy.
• Renal function parameters: eGFR and 24 h-UP.
• Other clinical and biochemical indicators: neutrophil count, lymphocyte count, platelet count, triglyceride (TG) level, high-density lipoprotein (HDL) level, blood pressure, and fasting blood glucose (FBG).
• Pathological features from biopsy specimens: the presence or absence of K-W nodules, capillary aneurysmal dilatation, fibrin cap, capsular drop, and crescent-shaped changes; as well as the severity of tubular atrophy, interstitial fibrosis, and arteriolar hyalinosis. All pathological assessments were independently reviewed and confirmed by at least 2 professional pathologists.

The following composite indices were calculated for the study:

$$\mathrm{AIP}=\log10\;\left(\mathrm{TG}/\mathrm{HDL}\right)$$ (1)

$$\mathrm{TyG}\;\text{index}=\mathrm{Ln}\;\left(\mathrm{TG}\times \mathrm{FBG}/2\right)$$ (2)

$$\begin{array}{c} \begin{array}{c} \text{ePWV}\;\left(m/s\right)\;\text{was calculated}\;\mathrm{as}:9.587-\left(0.402\times \mathrm{age}\right)\\ +\left(0.004560\times {\mathrm{age}}^{2}\right)-\left(0.002621\times {\mathrm{age}}^{2}\times \text{mean blood pressure}\;\left[\mathrm{MBP}\right]\right)\\ +\left(0.003176\times \mathrm{age}\times \mathrm{MBP}\right)-\left(0.01832\times \mathrm{MBP}\right),\text{where}\;\mathrm{MBP}\\ =\text{diastolic blood pressure}+0.4\\ \times \left(\text{systolic blood pressure}-\text{diastolic blood pressure}\right) \end{array} \end{array}$$ (3)

$$\begin{array}{c} \text{Systemic immune}-\text{inflammation index}\;\left(\mathrm{SII}\right)=\\ \text{platelet count}\times \left(\text{neutrophil count}/\text{lymphocyte count}\right) \end{array}$$ (4)

The primary composite kidney outcome was defined as a sustained decline in eGFR of ≥40% from baseline for over 4 weeks, progression to ESKD, or death from renal causes [35,36]. ESKD was defined as an eGFR of <15 ml/min/1.73 m^2^, or the need for long-term dialysis or kidney transplantation (either initiated or planned).

A total of 185 patients who met the eligibility criteria were finally included, with a follow-up loss rate of 13.15%. The sample size was estimated using Kendall’s guideline for multivariate analysis, which recommends 5 to 10 events per variable. Given the 17 variables included in this study, the estimated minimum required sample size ranged from 85 to 170. The actual sample size of 185, therefore, meets this common methodological requirement.

### Statistical analysis

All statistical analyses were performed using Python (version 3.13.2). Continuous variables that were normally distributed, as confirmed by tests for homogeneity of variances, are presented as mean ± standard deviation (SD) and were compared between groups using the independent samples *t* test. Continuous variables that did not follow a normal distribution are expressed as median with interquartile range and were compared using the Mann–Whitney *U* test. Categorical variables are summarized as frequencies and percentages (*n*, %) and were analyzed using the chi-square test to assess differences between groups. The primary evaluation metric was AUC, which measures the model’s overall discriminative ability across threshold settings. Additionally, accuracy, precision, recall, F1-score, and PR-AUC were reported to provide a complementary view of classification performance, particularly under class-imbalanced conditions. All probability-based metrics (ROC-AUC and PR-AUC) were computed using the model’s posterior probability outputs, whereas classification metrics were derived from binary predictions obtained at a fixed threshold. The PR-AUC is especially sensitive to model performance when the positive class is underrepresented.

### Feature selection

Predictors of the 1-year composite renal outcome in DKD patients were initially identified using univariate logistic regression analysis. Subsequently, the LASSO logistic regression was applied to further refine and compress the feature set identified from the univariate analysis. The optimal regularization parameter (*α*) was determined through 5-fold cross-validation. Features with nonzero coefficients were retained for the subsequent model development.

### Multimodel comparison and predictive model development

Based on the selected features, 6 distinct machine learning models were developed: multivariable logistic regression, RF, XGBoost, LightGBM, naive Bayes, and multilayer perceptron. During the data preprocessing phase, categorical variables were subjected to one-hot encoding, while continuous variables were standardized (*Z*-score normalization). The dataset was then stratified and randomly partitioned into a training set and a testing set at a ratio of 7:3.

Hyperparameter optimization was performed using 5-fold cross-validation on the training set. The ROC-AUC served as the primary metric for model evaluation. Model performance was comprehensively assessed using multiple additional metrics, including precision, recall, and the F1-score. The final classification threshold was determined based on the optimal F1-score. All analytical procedures incorporated considerations for class imbalance and implemented corresponding strategies to mitigate overfitting. Following a comprehensive evaluation of all metrics, the best-performing algorithm was selected for the final predictive model.

A heatmap for visualizing pathological image recognition features was generated by computing a weighted sum of the activations from the final convolutional layer of the model and the corresponding classification weights.

### Training and internal validation of the multimodal model

The final predictive model was developed using the top-performing algorithm identified in the previous step, which was ultimately determined to be the RF algorithm. While maintaining a consistent RF architecture, we constructed 3 distinct modeling workflows: a clinical model, a stage-stratified model (where pathological variables extracted by ResNet18 were input into the RF), and a fusion model (which combined pathological probabilities with clinical variables as inputs to the RF). These models were systematically compared using multidimensional evaluation metrics and stability assessments.

### Data processing

The internal cohort of 185 cases was split at the patient level in an 8:2 stratified ratio (stratified by the occurrence of the 1-year composite renal outcome). From 50 random seeds (1 to 50), the split that yielded the most balanced distribution of key variables—based on the lowest overall standardized mean difference—was selected to mitigate distributional bias introduced by random partitioning. The image model was trained exclusively on the internal training set, while the internal validation set was used for early stopping and selection of optimal weights based on AUC. During the RF training phase, only samples with complete clinical variables and outcome data were retained (cases with missing values were directly excluded) to avoid bias from inconsistent imputation. All features and labels were aligned at the patient level prior to being input into the model for training.

### Task definition and labeling

This study involves 2 distinct prediction tasks: (a) image-based prediction of pathological phenotypes: detection of crescent formation (*y*_c_) and interstitial fibrosis (*y*_f_); (b) clinical/fusion-model prediction of prognostic outcome: prediction of the composite renal outcome. Both tasks are formulated as binary classification problems, with labels derived directly from structured records. The first-level task focuses on evaluating the model’s ability to extract and discriminate morphological information from pathology images, while the second-level task assesses the combined contribution of pathological features and clinical variables in predicting patient prognosis.

### Image model (ResNet18)

The image model employed ResNet18 as the feature encoder, with its original classification head replaced by a 2-layer fully connected network. When available, the model was initialized with ImageNet-pretrained weights; otherwise, training proceeded from random initialization. Let the feature vector from the penultimate layer be denoted as $h\in R^{512}$. The classification head is defined as follows:

$$z=W2\;\sigma \left(W1\;h+b1\right)+b2$$ (5)

where *σ*(·) denotes the ReLU activation function, and a dropout rate of *p* = 0.5 was applied. The class probabilities are obtained via softmax normalization:

$$p=\text{softmax}\left(z\right)$$ (6)

For the binary classification task, let *ŷ_i_* = *P* (*y_i_* = 1 | *x_i_*) denote the predicted probability of the positive class. The model is optimized using the binary cross-entropy loss function:

$$\begin{array}{c} L_{\mathrm{img}}=-\frac{1}{N}\sum_{i=1}^{N}\left[y_{i}\log{\hat{p}}_{i}+\left(1-y_{i}\right)\log\left(1-{\hat{p}}_{i}\right)\right] \end{array}$$ (7)

The input image size was set to 224 × 224 pixels. During training, data augmentation was applied using RandomResizedCrop, random horizontal flip, random rotation, and ColorJitter. For the validation phase, images were processed using Resize(256) followed by CenterCrop(224). All image tensors were normalized with the ImageNet mean and standard deviation to maintain statistical consistency with the pretrained weights.

The optimizer used was Adam with a learning rate of 1 × 10^−4^, a weight decay of 1 × 10^−4^, and a batch size of 16. Training was run for a maximum of 15 epochs, with early stopping and model selection based on the internal validation AUC (patience = 4). This early stopping strategy helps mitigate overfitting in small-sample settings while avoiding unnecessary iterations.

### Patient-level aggregation

For each patient, the image model outputs a per-slide probability *p_i_*. The patient-level mean probability is defined as:

$$\bar{p}=\frac{1}{n}\sum_{i=1}^{n}p_{i}$$ (8)

Multiple patient-level aggregation statistics—including the maximum, median, standard deviation, minimum, positive-class ratio, and entropy—were computed to characterize the central tendency, dispersion, and uncertainty of the image-level probability distribution within each patient. These aggregated metrics compress multiple image observations at the patient level, thereby reducing the influence of random error from individual slides on downstream classifiers.

The patient-level binary output for each pathological phenotype was determined via majority voting. The thresholds for crescents (*n* = 3) and fibrosis (*n* = 5) were respectively defined as:

$${\hat{y}}_{c}=I\left(\sum_{i=1}^{3}I\left(p_{i}\geq 0.5\right)\geq 2\right)$$ (9)

$${\hat{y}}_{f}=I\left(\sum_{i=1}^{5}I\left(p_{i}\geq 0.5\right)\geq 3\right)$$ (10)

Both the stage-stratified model and the fusion model used patient-level probability features for downstream modeling, while the majority-vote binary outputs served only as auxiliary statistics. The above design may introduce variability in internal validation performance in small-sample settings; therefore, the results should be interpreted in conjunction with external validation and stability assessments.

### RF model and 3 modeling pipelines

The RF model is composed of multiple decision trees, employing bootstrap aggregation and random feature subset selection to reduce variance. To mitigate the influence of class imbalance on the splitting criterion, “class_weight='balanced'” was applied, and the Gini impurity (the default in scikit-learn) was used as the splitting criterion. The input features for the 3 models were defined as follows:

$$X_{\text{clinical}}=\left[\text{ePWV},\;\mathrm{SII},\;24\;h-\mathrm{UP},\;\text{eGFR}\right]$$ (11)

$$X_{\text{stage}}=\left[\hat{p}c,\;\hat{p}f,\;\text{ePWV},\;\mathrm{SII},\;24\;h-\mathrm{UP},\;\text{eGFR}\right]$$ (12)

$$X_{\text{fusion}}=\left[\bar{p}c,\;\bar{p}f,\;\text{ePWV},\;\mathrm{SII},\;24\;h-\mathrm{UP},\;\text{eGFR}\right]$$ (13)

For the clinical and fusion models, fixed hyperparameters were used: n_estimators = 300, max_depth = 4, min_samples_leaf = 6, max_features = sqrt, class_weight = balanced, and random_state = 42. To further suppress overfitting, stronger regularization was applied to the fusion model: n_estimators = 60, max_depth = 1, min_samples_leaf = 20, max_features = sqrt, and class_weight = balanced, random_state = 42. The stage-stratified model employed tuned hyperparameters: n_estimators = 200, max_depth = 8, min_samples_leaf = 1, max_features = 0.7, class_weight = balanced, and random_state = 8,410.

### External validation of the multimodal model

Data from 137 patients treated at Hebei University Affiliated Hospital between 2005 June 1 and 2024 May 30, who met the inclusion and exclusion criteria, were selected for external validation. The external validation data were not used in any training or early-stopping procedures. There was no patient-level overlap between the internal and external cohorts; the external set was employed exclusively for final model evaluation.

As suggested by Riley et al. [37], the 95% CI width for the AUC should be kept within 0.10, which translates to a standard error of approximately:

$$\mathrm{SE}\left(C\right)\leq \frac{0.10}{2\times 1.96}=0.0255$$ (14)

Substituting:

C=0.923,ϕ=0.486 (15)

Iterative estimation using the Newcombe formula yielded:

N≈125 (16)

### Stability assessment

To quantify model stability, the stage-stratified, clinical, and fusion models were each evaluated using repeated random splits and cross-validation. For the internal data, 20 different random seeds were used to perform stratified 8:2 splits. Additionally, 5-fold stratified cross-validation was applied to obtain the distribution of AUC values. To further assess robustness, both internal and external validation AUCs were subjected to 2,000 bootstrap resamples, with the corresponding 95% CIs reported.

## Data Availability

The analysis code and model definitions are available from the GitHub repository at https://github.com/giselle316/DKD_predictor. The whole-slide image data necessary for model development can be retrieved from the Zenodo repository at https://doi.org/10.5281/zenodo.18534147. Both resources are available for download to enable local deployment.
